# Complications associated with intestinal infusion therapies in patients with Parkinson’s disease: a single-center retrospective study and 15-year experience

**DOI:** 10.3389/fneur.2025.1547557

**Published:** 2025-05-14

**Authors:** Igor Straka, Zuzana Andre, Zuzana Kosutzka, Karin Gmitterova, Milos Stevove, Zuzana Durkovicova, Radovan Juricek, Peter Valkovic, Michal Minar

**Affiliations:** ^1^2^nd^ Department of Neurology, Comenius University Bratislava, Faculty of Medicine, University Hospital Bratislava, Bratislava, Slovakia; ^2^Department of Neurology, Slovak Medical University, Faculty of Medicine, University Hospital Bratislava, Bratislava, Slovakia; ^3^3^rd^ Department of Internal Medicine, Comenius University Bratislava, Faculty of Medicine, University Hospital Bratislava, Bratislava, Slovakia; ^4^Department of Gastroenterology, Hospital Bory, Bratislava, Slovakia; ^5^Institute of Normal and Pathological Physiology, Centre of Experimental Medicine, Slovak Academy of Sciences, Bratislava, Slovakia

**Keywords:** advanced Parkinson’s disease, adverse events, gastrojejunostomy, levodopa/carbidopa intestinal gel (LCIG), levodopa/entacapone/carbidopa intestinal gel (LECIG)

## Abstract

**Introduction:**

Parkinson’s disease (PD) is a progressive neurodegenerative disorder, in advanced stages characterized by motor and non-motor fluctuations, significantly impacting patients’ quality of life (QoL). Advanced therapies, such as levodopa/carbidopa intestinal gel or carbidopa/levodopa enteral suspension (LCIG/CLES) and levodopa/entacapone/carbidopa intestinal gel (LECIG), offer continuous levodopa administration to reduce fluctuations and improve QoL. However, these therapies require invasive percutaneous endoscopic gastrostomy with jejunal extension (PEG-J), which can lead to complications. This study aimed to analyze the incidence of complications related to gastrojejunostomy in patients treated with LCIG/CLES or LECIG and their impact on therapy outcomes.

**Methods:**

This retrospective study included PD patients treated with LCIG/CLES or LECIG at our center over 15 years. Patients were included if they had a PEG-J inserted and had been on LCIG/CLES or LECIG for at least 3 months. Complications were analyzed to identify trends and practical solutions for management.

**Results:**

Of 111 PEG-J insertions, we analyzed 106 patients treated with LCIG/CLES or LECIG. A total of 77.4% experienced at least one adverse event (AE), predominantly device-related (69.8%). Common complications included knotting (24.4%), disconnection (22.8%), and occlusion (17.1%) of the inner tube. Serious AEs were rare but included three deaths within 30 days post-procedure, severe skin phlegmon in two patients, and severe gastrointestinal discomfort in one patient. The duration of PEG-J significantly correlated with AEs.

**Conclusion:**

Gastrojejunostomy-related AEs in LCIG/CLES and LECIG therapies are common but generally manageable with proper intervention. Serious complications are rare, with less than 10% discontinuing treatment due to dissatisfaction.

## Introduction

Parkinson’s disease (PD) is a progressive, debilitating, multisystem neurodegenerative disorder that, in its advanced stages, is characterized by both motor and non-motor fluctuations. These complications often include dyskinesias, which are common side effects of long-term oral levodopa therapy and continuous neurodegeneration. These motor complications not only complicate PD management, but also significantly impact patients’ quality of life (QoL), caregivers’ burden, social and healthcare systems (1). When oral treatments fail to provide optimal motor control, advanced therapeutic strategies (device-aided therapies) such as surgical methods (e.g., deep brain stimulation – DBS), intestinal infusion (pump) therapies (levodopa/carbidopa intestinal gel – LCIG or CLES in the US – carbidopa/levodopa enteral suspension, and levodopa/entacapone/carbidopa intestinal gel – LECIG), continuous subcutaneous apomorphine infusion, and novel subcutaneous infusions of levodopa/carbidopa or foslevodopa/foscarbidopa become necessary (2).

The advantage of LCIG/CLES and LECIG treatments is the continuous administration of levodopa through a percutaneous endoscopic gastrostomy with jejunal extension (PEG-J) throughout the day, ensuring a constant plasma levodopa level, and significantly reducing fluctuations and improving QoL. This therapy involves direct application of gel infusion to the proximal jejunum, bypassing the stomach (3). In many studies, LCIG/CLES significantly reduced “OFF”-time, increased “ON”-time, and improved QoL and activities of daily living (4–8). The effectiveness of LECIG infusion has also been confirmed in the first studies. This treatment has the advantage of using a smaller, lighter pump, and entacapone provides higher bioavailability of levodopa from the infusion (1, 9).

The disadvantages of LCIG/CLES and LECIG therapies are the invasiveness associated with PEG-J insertion and the resulting complications. Adverse events (AEs) are common, reported in 54.5 to 94% of patients. Up to 76% of AEs are related to the PEG-J procedure, device, or gastrointestinal problems, and 53% are serious (4, 5, 10–12). The summary of treatment-associated AEs is shown in Table 1 (10, 13). Stoma (skin) related AEs are secretions, infections or abdominal cellulite (14, 15). Patients treated with intestinal infusion therapies have a higher rate of peripheral polyneuropathy, probably due to levodopa metabolic products (16). Despite these AEs, treatment is very effective and only a small percentage of patients wish to discontinue treatment.

**Table 1:** Complications associated with PEG-J insertion.

Complications associated with PEG-J insertion		Complications associated with device	Complications associated with levodopa treatment
Minor:	Major:	Dislocations of tube	Hallucinations, psychosis
Local infections dermatitis	Aspiration pneumonia	Disconnections of tube	Insomnia, daytime sleepiness
Pneumoperitoneum	Hemorrhage	Occlusion of tube	Anorexia, nausea
Stoma leakage	Buried bumper syndrome	Knotting of tube	Palpitations
Inadvertent PEG-J removal	Peritonitis	Leakage of tube	Orthostatic hypotension
Gastric outlet obstruction	Perforation of bowel	Malfunction of connectors	Polyneuropathy
	Necrotizing fasciitis	Pump failure	
	Metastatic seeding		

PEG-J, percutaneous endoscopis gastrostomy with jejunal extension.

Although LCIG/CLES has been available in Europe since 2004, specific criteria for the selection of suitable candidates remain undefined. Establishing these criteria is particularly important in light of new treatment options, especially with the development of subcutaneous foslevodopa or levodopa treatment. In most countries, patients who are unsuitable or refuse DBS are subsequently referred to infusion therapy (10, 17, 18).

In Slovakia, treatment with DBS, intestinal infusion therapies (LCIG/CLES and LECIG) and subcutaneous apomorphine infusion is currently available. Currently, 96 patients are being treated with DBS and 49 patients are being treated with intestinal infusion therapies at our center. However, this ratio varies according to each center’s traditions, experience, and availability of each therapy. As part of the indication process, the first decision is whether the patient is indicated for DBS implantation. If the patient is not indicated for this treatment (due to cognitive impairment, postural instability or ON-freezing of gait, or severe speech impairment), or based on patient preference, infusion therapies are considered. Due to the side effects of apomorphine, we usually use this treatment for a shorter period of time, e.g., before DBS or intestinal infusion therapies are indicated (in Slovakia, these therapies require the approval of the health insurance company). For intestinal infusion therapies, the decision for LCIG/CLES or LECIG is based on tolerance to catechol-O-methyltransferase inhibitors or neuropsychiatric profile. Although there are no recommendations yet, in patients with a history of hallucinations or other psychotic reactions, we prefer to treat with LCIG/CLES.

Our study aims to retrospectively analyze complications related to PEG-J in patients with Parkinson’s disease treated with LCIG/CLES and LECIG at our Center for Movement Disorders.

## Methods

We included patients who met the UK-PD Society Brain Bank Criteria (19) and the MDS clinical diagnostic criteria for PD (20) and had been treated with LCIG/CLES or LECIG at the Center for Movement Disorders of the 2nd Department of Neurology, Faculty of Medicine, Comenius University Bratislava, University Hospital Bratislava – Derer’s Hospital, Bratislava, Slovakia. All PEG-J insertions and revisions were performed at the endoscopic unit of the 3rd Department of Internal Medicine, Faculty of Medicine, Comenius University Bratislava, University Hospital Bratislava – Derer’s Hospital, Bratislava, Slovakia. All gastroenterologists involved were certified in endoscopy with over 5 years of experience.

We enrolled patients who had undergone PEG-J insertion between November 2009 and May 2024, with a minimum treatment duration of 3 months using LCIG/CLES or LECIG. The majority of patients underwent nasojejunal tube testing prior to definitive PEG-J insertion (testing was no longer required after 2022). Endoscopic and neurological findings were retrieved from the Hospital Information System Medea 13.2.1 and analyzed for reported gastrojejunostomy, skin and device related AEs. Levodopa-induced complications, such as peripheral polyneuropathy, were assessed using nerve conduction studies, but only in patients presenting clinical symptoms of polyneuropathy (e.g., reduced tactile sensation, muscle weakness, or paresthesias). Among the serious AEs, we included 30-day mortality following PEG-J insertion, inflammatory and bleeding complications related to PEG-J insertion, buried bumper syndrome, severe skin phlegmon, severe gastrointestinal discomfort and severe acute peripheral polyneuropathy. We analyzed all patients who underwent PEG-J insertion for LCIG/CLES (since 2009) and LECIG (since 2022) treatments. This study complies with the Declaration of Helsinki, approved by the Ethics Committee of the Derer’s Hospital, under approval number 13/2021.

Adverse events (AEs) were recorded retrospectively based on clinical documentation from routine follow-up visits. PEG-J related AEs were identified either through spontaneous patient reports (e.g., worsening of clinical condition, leakage, discomfort, accidental removal) or through direct physical examination. The PEG-J stoma site was routinely assessed at each visit for signs of infection, local irritation, or mechanical complications. Peripheral polyneuropathy was actively screened for as part of standard neurological examination, based on typical clinical symptoms and signs. Psychotic symptoms were recorded based on reports from patients or caregivers, or noted in medical documentation.

Demographic and clinical parameters were analyzed using descriptive statistics. The level of variables was characterized by the median and the basic measures of variability by the interquartile range, minimum and maximum values. The strength of the relationship between parameters was measured by Spearman’s rank correlation coefficient (r_S_) and correlation ratio eta (*η*).

## Results

Between 2009 and 2014, we performed 111 PEG-J insertions for LCIG/CLES (97 patients) and LECIG (14 patients) treatments. Two patients were on the treatment less than 3 months. Thirty-day mortality after the procedure was 2.70% (three patients: two patients died from aspiration pneumonia, and one patient died from massive pulmonary embolism). Patients with thirty-day post-procedure mortality and two patients treated for less than 3 months were not included in the analysis.

We included a total of 106 patients in the further analysis. Two patients were switched from LCIG/CLES to LECIG treatment due to the need for higher doses and the insufficient effect of LCIG/CLES. In our cohort, two patients received a combination of DBS and LCIG/CLES treatment. In four cases, the procedure was carried out in cooperation with a surgeon due to the absence of diaphanoscopy.

The basic demographic and clinical data are presented in Table 2. Currently, 49 patients are treated with intestinal infusion therapy (35 on LCIG/CLES treatment and 14 on LECIG treatment). Fifty-seven patients discontinued the treatment – 38 patients died, seven patients decided to discontinue LCIG/CLES treatment at their own request (four due to lack of effectiveness, two due to repeated gastrofibroscopies, and one due to difficulty operating the device after 9 years of treatment), seven due to non-cooperation related to severe dementia, two because of skin phlegmon in the PEG-J insertion area, two due to buried bumper syndrome, and one because of severe gastrointestinal discomfort. The strict therapy retention rate, defined as the proportion of patients still actively receiving treatment at the time of analysis, was 46.2% (49/106 patients). When including patients who remained on therapy until death, the retention rate was 82.1% (87/106 patients), reflecting those who did not discontinue the therapy due to AEs or dissatisfaction. In our cohort, only 12 patients (11.3%) discontinued therapy due to AEs, device-related issues, or lack of effectiveness.

**Table 2:** Basic demographic and clinical data.

Age at PEG-J initiation		69.50 (65.00; 74.00 / 54.00–82.00)
PD duration at PEG-J initiation (years)		11.00 (8.00; 14.25 / 3.00–21.00)
Sex	Female Male	38 68
Hoehn and Yahr at initiation		3.0 (3.0–3.0 / 2.50–5.00)
Duration of LCIG/LECIG treatment included in the analysis (all patients)	Months Years	30.00 (10.75; 60.00 / 3.00–137.00) 2.50 (0.90; 5.00 / 0.25–11.42)
Duration of LCIG/LECIG treatment (currently on the treatment)	Months Years	23.00 (6.00; 59.25 / 3.00–110.00) 1.92 (0.50; 4.94 / 0.25–9.17)
Duration of LCIG/LECIG treatment (terminated treatment)	Months Years	37.50 (13.00; 61.75 / 3.00–137.00) 3.13 (1.08; 5.15 / 0.25–11.42)
Duration of LCIG/LECIG treatment (terminated treatment due to death)	Months Years	45.50 (27.75; 64.50 / 4.00–134) 3.80 (2.31; 5.38 / 0.33–11.17)
Duration of LCIG/LECIG treatment (terminated due to discontinuation of treatment)		13.50 (6.25; 40.00 / 3.00–137) 1.13 (0.52; 3.33 / 0.25–11.42)
Number of complications per patient associated with device		1.00 (0.00; 3.00 / 0.00–11.00)
Duration to the first complication (months) associated with device		6.50 (2.00; 22.50 / 0.00–52.00)

Median (IQR – 25. percentile; 75. percentile / MIN – MAX). PD, Parkinson’s disease; LCIG – levodopa/carbidopa intestinal gel; LECIG, levodopa/entacapone/carbidopa intestinal gel; PEG-J, percutaneous endoscopis gastrostomy with jejunal extension.

The overview of PEG-J insertions and revisions is presented in Table 3. In our cohort, 82 patients (77.36%) had at least one AE associated with PEG-J. Seventy-four patients (69.81%) had AEs associated with the device. The median number of device-associated complications was one (Table 4), and the median time to the onset of complications was 6.5 months after PEG-J insertion. A total of 193 PEG-J revisions were performed: 47 (24.35%) cases for knotting of the inner tube, 44 cases (22.80%) for disconnection of the inner tube, 33 cases (17.10%) for occlusion of the inner tube, 28 cases (14.51%) for dislocation of the inner tube, 15 cases (7.77%) for leakage of the tube, 15 cases (7.77%) for inadvertent PEG-J removal, nine cases (4.66%) for malfunction of connectors, and two cases (1.04%) for buried bumper syndrome. In terms of individual patients, the most commonly reported complications were disconnection of the inner tube in 31 patients (29.25%), occlusion in 27 patients (25.47%), and knotting of the inner tube in 26 patients (24.53%) (Table 5). Routine tube replacements were not performed; all revisions were prompted by specific complications such as knotting, disconnection, occlusion, or other device-related issues.

**Table 3:** Number of PEG-J insertions and revisions.

Year	2009	2010	2011	2012	2013	2014	2015	2016	2017	2018	2019	2020	2021	2022	2023	2024
Currently treated patients	1	3	8	12	16	19	23	21	25	26	31	33	32	36	42	49
PEG-J insertions	1	2	5	6	6	6	5	4	6	8	12	8	3	10	15	14
PEG-J revisions	0	0	2	5	3	13	18	13	12	18	22	21	19	18	19	10

PEG-J, percutaneous endoscopis gastrostomy with jejunal extension.

**Table 4:** Number of adverse events associated with device in our patients.

Number of AEs	Number of patients (%)
0	32 (30.18)
1	24 (22.64)
2	23 (21.70)
3	12 (11.32)
4	7 (6.60)
5	2 (1.89)
6	1 (0.94)
7	1 (0.94)
8	2 (1.89)
9	1 (0.94)
11	1 (0.94)

AE, adverse event.

**Table 5:** Adverse events associated with device.

Adverse event	Number of events (%)	Number of patients (%)	Number of patients with 1 AE	Number of patients with 2 AEs	Number of patients with 3 AEs	Number of patients with ≥ 4 AEs
Knotting of inner tube	47 (24.35)	26 (24.53)	17	3	4	2
Disconnection of inner tube	44 (22.80)	31 (29.25)	21	8	1	1
Occlusion of inner tube	33 (17.10)	27 (25.47)	21	6	0	0
Dislocation of inner tube	28 (14.51)	23 (21.70)	21	2	1	0
Leakage of tube	15 (7.77)	15 (14.15)	15	0	0	0
Inadvertent PEG-J removal	15 (7.77)	13 (12.26)	12	0	1	0
Malfunction of connectors	9 (4.66)	9 (8.49)	9	0	0	0
Buried bumper syndrome	2 (1.04)	2 (1.89)	2	0	0	0

AE, adverse event; PEG-J, percutaneous endoscopis gastrostomy with jejunal extension.

Local skin AEs associated with PEG-J occurred in 55 patients (51.89%); 18 patients (16.98%) had granuloma, 28 patients (26.42%) had dermatitis, and 13 patients (12.26%) had infections, two female patients (1.89%) had serious skin phlegmon requiring PEG-J removal.

Other AEs associated with PEG-J insertion included abdominal abscess with subsequent puncture and drainage in one patient (0.94%), gastric ulcer with colitis and subsequent surgical treatment and therapy with proton pump inhibitors (0.94%), and one patient who had severe bleeding from a gastric ulcer with hemorrhagic shock. In these three cases, it was not necessary to stop the treatment. One patient (0.94%) developed severe gastrointestinal discomfort immediately after PEG-J insertion, which led to discontinuation of the treatment.

Adverse events significantly correlated with duration of PEG-J initiation (r_S_ = 0.589, *p* < 0.001), but we did not find a correlation between AEs and age (r_S_ = −0.073, *p* = 0.106) or gender (*η* = 0.038, *p* = 0.699).

Most AEs were managed by gastrofibroscopic revision. Serious AEs occurred in our cohort:

- three patients died within 30 days after PEG-J initiation,
- skin phlegmon in two patients,
- two patients had buried bumper syndrome,
- one patient had gastric ulcer with colitis,
- one patient had gastric ulcer with severe bleeding,
- two patients had severe gastrointestinal discomfort.

**Figure 1:**
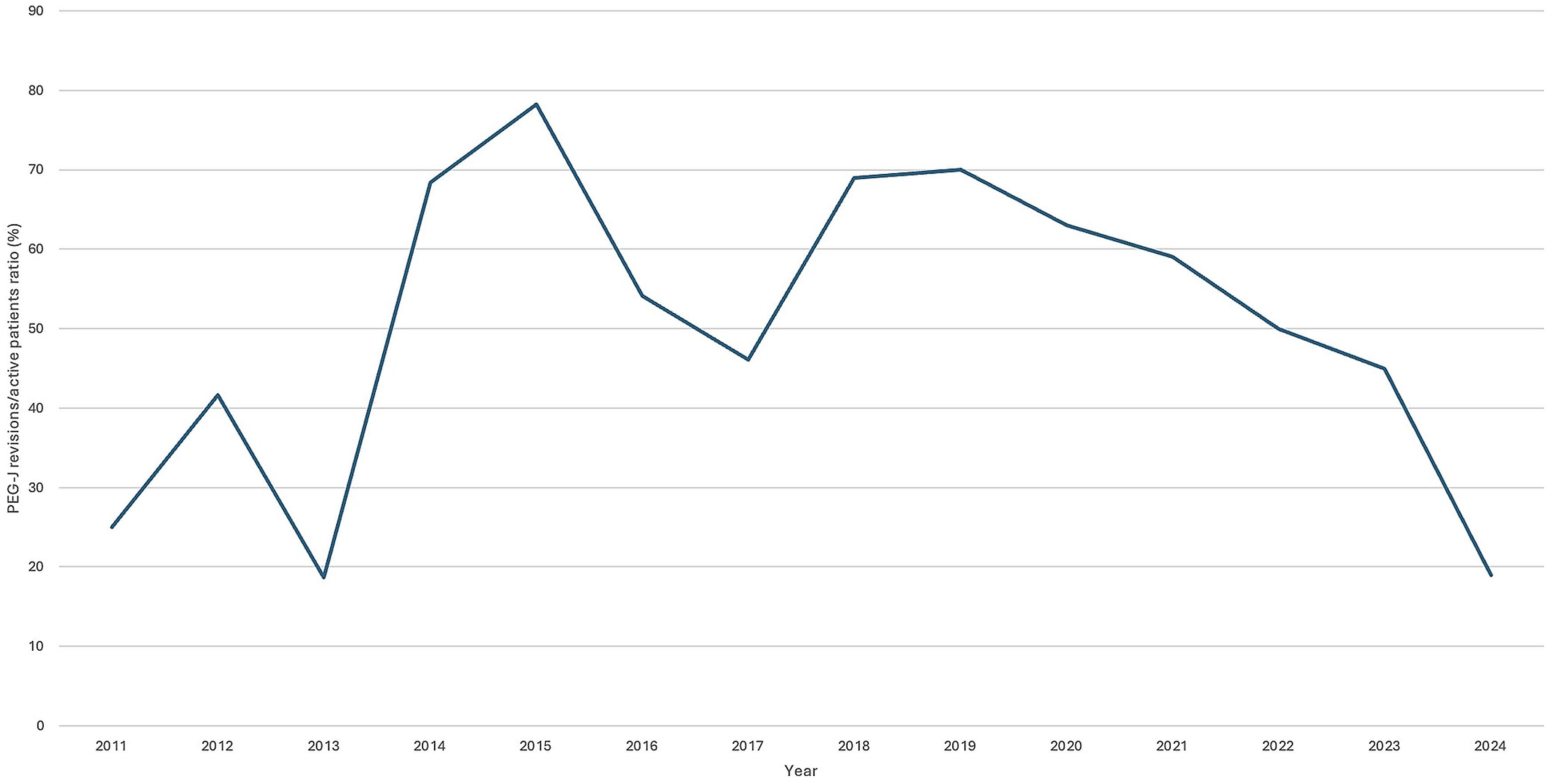
Change in the ratio of PEG-J revisions to treated patients over the years.

We analyzed the ratio of PEG-J revisions to currently treated patients for each year (Figure 1). From 2019 onwards, a gradually decreasing trend in the occurrence of complications has been observed. This trend may be potentially attributed to the growing experience of the center with the therapy. However, it is equally important to highlight that many PEG-J-related complications are unpredictable and may not directly correlate with the center’s learning curve.

In our cohort, polyneuropathy was diagnosed in 24 patients (22.64%) based on clinical symptoms and was subsequently confirmed by nerve conduction studies. All these patients were on LCIG/CLES treatment. One patient developed severe acute demyelinating sensory polyneuropathy. In this patient, we temporarily discontinued LCIG/CLES treatment and subsequently reinitiated it after vitamin therapy.

Twenty-one patients (19.81%) experienced psychosis during treatment. Psychosis occurred more frequently in patients with dementia and, given the persistence of psychotic symptoms in some PD patients with dementia, this was a reason for discontinuing treatment (as mentioned above, seven patients with dementia discontinued the treatment).

## Discussion

Intestinal infusion therapies with LCIG/CLES and LECIG are well-established methods for managing advanced PD, offering benefits such as reducing OFF states, improving some non-motor symptoms, and enhancing QoL (21, 22). Our study presents data from a single center on complications related to gastrojejunostomy and the infusion device.

Our findings indicate that gastrojejunostomy-related AEs are common but generally manageable. In our cohort, 77.4% of patients experienced at least one AE, predominantly device-related (69.8%), with complication rates increasing over time. These findings are slightly higher than in previous studies, where complication rates varied depending on follow-up duration: 35.1% at 0.91 years (23) and 63.5% at 13 years (10). This may be related to the longer duration of the study (15 years) and the fact that all complications were reported to our Center for movement disorders, after which we subsequently contacted the endoscopy unit. All patients were managed by the same endoscopy unit. The total number of complications associated with using PEG-J for LCIG/CLES or LECIG administration is higher in patients with PD than in patients receiving nutritional PEG (24), but the rate of serious complications is similar (25, 26).

In our study, the mean number of device associated complications was equal to 1.82. In other studies, the mean varied between 0.48 and 6.1 (4, 6, 10–12, 23, 27, 28). The most common causes of device complications vary across studies, including dislocations (6, 11, 12), accidental removals (10, 26, 28), and occlusions (23). In our cohort, the most common device relate complications were knotting, disconnection, and occlusion of the inner tube.

Two of our patients (1.89%) had buried bumper syndrome, leading to discontinuation of treatment in both cases. Prevention strategies for this syndrome include ensuring that the internal bumper is placed gently against the stomach wall without excessive tension, regularly pulling of the PEG tube to prevent tissue overgrowth around the bumper, and routinely checking for excessive tension, redness or pain at the tube site (29). The frequency of buried bumper syndrome ranges from 0.3 to 2.4% (30).

No deaths directly related to PEG-J insertion were present in our study. Three patients (2.7%) died within 30 days of the procedure, with no direct link to the procedure of PEG-J insertion or LCIG/CLES and LECIG therapy. In our cohort, two patients died from aspiration pneumonia, and one patient died from massive pulmonary embolism. This 30-day mortality rate is comparable to other conditions requiring PEG insertion. Higher mortality rates after PEG insertion are seen in severe conditions such as strokes or oncological diseases (31). Direct life-threatening conditions, such as severe peritonitis, intestinal perforation (32), and liver damage (33), have been reported in the literature. Only 0.5% of deaths are related to the LCIG/CLES treatment system and are typically caused by PD complications (such as pneumonia) rather than AEs (11).

Twenty patients (18.9%) discontinued treatment, and only seven patients (6.6%) discontinued treatment due to dissatisfaction. This proportion is similar compared to the literature, which reports discontinuation rates of 3.3 to 25.7% (11, 26, 34). Most patients tend to discontinue due to inefficacy, disease progression (e.g., dementia, comorbidities) (34), recurrent device complications (26), or severe polyneuropathy (12). In our Center for Movement Disorders, other reasons for discontinuation were also dementia or repeated gastrofibroscopy and its associated discomfort. However, no patient discontinued treatment due to severe peripheral polyneuropathy. In our cohort, polyneuropathy occurred in 22.6% of patients, with only one patient experiencing severe polyneuropathy, which did not require treatment discontinuation. The prevalence of peripheral polyneuropathy was comparable to that reported in other studies (35–37).

Skin AEs were within expected ranges, with dermatitis, granulomas, and infections being the most common. Two patient required PEG-J removal due to severe skin phlegmon. We did not report any cases of pneumoperitoneum, likely because we do not perform standard imaging after PEG-J insertion, potentially leading to its underestimation (25, 26).

Psychosis occurs in 3 to 30% of patients with PD (38). In patients receiving intestinal infusion therapies, the prevalence ranges from 10 to 27% (28, 39). In our cohort, psychotic symptoms were observed in 19.8% of patients.

A recent Slovak study (24) reported a higher incidence of complications in PD patients treated with LCIG via PEG-J compared to patients receiving standard nutritional PEG. This aligns with our findings and supports the observation that PEG-J carries specific risks in PD patients beyond those seen in other indications. Compared to major European studies (5, 6), our complication rates are slightly higher, likely due to our longer follow-up (up to 15 years) and systematic reporting through a single center. However, the nature and distribution of adverse events, as well as the low discontinuation rate due to dissatisfaction, remain consistent with those studies.

The incidence of AEs in our patients compared to other studies is shown in Table 6.

**Table 6:** Incidence of AEs in our patients compared with other studies.

Adverse event	Incidence in our study (%)	Literature range (%)
Patients with AEs	77.4	48.6–95 (6, 10–12, 23, 26–28, 40)
**Total skin AEs**	**51.9**	**36.4–69.8** (12, 23, 26, 27)
Granuloma	17.0	10–34.9 (10, 12, 24, 28)
Infection	12.3	13.3–45 (10, 12, 23, 24, 27, 40)
Dermatitis	26.4	20–31.2 (10, 12, 26)
Abdominal discomfort	0.9	3.1–15.9 (10, 12, 27)
Peritonitis	0	0–4.8 (6, 10, 23, 26, 28)
**Device AEs**	**69.8**	**35.1–92** (6, 10, 12, 23, 26, 40)
Knotting of inner tube	24.5	5.4–7.8 (23, 28)
Disconnection of inner tube	29.2	10.8–38 (23, 26)
Occlusion of inner tube	25.4	10.8–31,7 (10, 23, 26)
Dislocation of inner tube	21.7	7–40 (6, 10, 12, 26, 28)
Leakage of tube	14.1	2.7–20 (10, 12, 23, 24, 26)
Inadvertent PEG-J removal	12.2	3.3–12.6 (12, 23, 28)
Malfunction of connectors	8.5	2.9–6 (6, 28)
Buried bumper syndrome	1.9	1–11.1 (10, 12, 24, 26, 28)

AE, adverse event; PEG-J, percutaneous endoscopis gastrostomy with jejunal extension.

Based on our findings and experience, we suggest several measures that may help reduce therapy discontinuation in clinical practice: routine monitoring of the PEG-J site at each visit, close collaboration between neurology and endoscopy teams to minimize unnecessary endoscopic interventions, active screening and management of motor and non-motor complications, ongoing education and support for patients and caregivers. Regular three-monthly follow-up visits, combined with the availability of a telephone helpline and e-mail contact, have contributed significantly to patient confidence and timely resolution of device-related or clinical problems.

This study summarizes a 15-year single-center experience with LCIG and LECIG therapies, focusing especially on complications related to PEG-J and the device. Our analysis offers a relatively detailed overview of device-related AEs, including the distribution and timing of specific complications. We also report a gradually decreasing trend in PEG-J revisions since 2019, which may reflect growing clinical experience with these therapies. In terms of treatment discontinuation, only a small proportion of patients stopped therapy due to dissatisfaction or AEs, which is comparable to previously published data. To our knowledge, this is the largest and longest observational study from our region focusing on real-world infusion-related complications in PD patients treated with LCIG/CLES and LECIG. The results support the importance of structured follow-up, caregiver education, and early response to complications in maintaining long-term therapy adherence. Future research should aim to compare complication profiles between LCIG/CLES and LECIG in larger, balanced cohorts, and to explore how emerging subcutaneous therapies may influence the role and indications of intestinal infusion therapies. It will also be important to evaluate, based on real-world data, what proportion of patients may switch from subcutaneous to intestinal infusion therapies, and for which clinical reasons – particularly due to skin-related AEs.

Our study’s retrospective design and potential data loss during long follow-up periods are limitations. Additionally, minor complications may have been underreported by patients. We did not compare the incidence of complications between LCIG/CLES and LECIG due to the disproportionate number of patients in each cohort and the availability of treatment in Slovakia (LCIG/CLES since 2009, LECIG since 2022). Another limitation may be the absence of a control group; however, standard nutritional PEG is typically performed in much more severe conditions than therapeutic PEG in PD, so these data would not be entirely comparable.

## Conclusion

Gastrojejunostomy-related AEs in LCIG/CLES and LECIG therapies are common but generally manageable with proper intervention. Serious complications are rare, and less than 10% discontinuing treatment due to dissatisfaction. Management strategies, including regular monitoring and patient education, are crucial for optimizing treatment outcomes. Early referral to specialists and personalized care are essential to enhance the effectiveness of advanced PD therapies (device-aided therapies).

### Our best practice in the care of PD patients on intestinal infusion therapies and perspectives

1. In our Center for Movement Disorders, teams have been formed for each of the device-aided therapies (DBS team, Infusion therapies team). However, all physicians at our center are experienced in all these modalities.
2. The initial indication process for infusion therapies, including thorough education, is performed in the outpatient clinic. The indication does not require testing of the therapy via a nasojejunal tube. In case of doubt, or if the patient wishes to try the treatment using a nasojejunal tube, the patient is admitted to our department.
3. For treatment initiation, the patient is admitted to our department. The treatment starts as soon as the patient arrives from the endoscopy unit. The patient is managed by a movement disorders specialist. The length of the hospital stay depends on the stabilization of the condition and any associated complications.
4. The patient is discharged home in the best possible motor condition. On discharge, detailed education for patient and family/caregivers is provided by the movement disorders specialist.
5. Patients have a telephone helpline or e-mail contact in case of complications or issues. All complications or issues are managed by a movement disorders specialist.
6. The patient’s first check-up at our center is no later than 4 weeks after discharge; if necessary, a check-up will be scheduled immediately.
7. Thereafter, the patient will undergo regular check-ups every 3 months; if necessary, a check-up will be arranged immediately.
8. The movement disorders nurse is involved in the education process and in the management of complications.

Management of PEG-J–related complications in our center follows a standardized, team-based approach. In cases of tube occlusion or disconnection, patients are typically referred for endoscopic assessment within 24–72 h, and oral pharmacotherapy is provided as needed during the interim period. Minor leakages or local skin issues are managed conservatively with local antiseptics and hygiene measures. Severe skin infections require systemic antibiotic therapy, in cooperation with a gastroenterologist, surgeon, or dermatologist. Inadvertent PEG-J removal is assessed on an individual basis. Patients are informed in their medical report that, in such an event, they should visit the nearest emergency department as soon as possible to have a urinary catheter inserted into the stoma site to preserve the stoma canal. If treatment continuation is appropriate, the endoscopy team will reintroduce the PEG-J. However, if there is a significant risk of repeated self-removal, reintroduction is not attempted.

New subcutaneous infusion therapies mark the beginning of a new era in the treatment of advanced PD. However, as these are newly developed therapies, data on their efficacy and safety from real clinical practice are still lacking. Further experience will demonstrate how they will impact the indication process for device-aided therapies. Nevertheless, we believe that intestinal therapies will continue to have an irreplaceable role. These therapies will remain an option for patients at more advanced stages of the disease or for those whose caregivers face motor difficulties, as they offer easier handling. Additionally, intestinal therapies seem to be a better choice for patients with cachexia, skin problems, or skin-related complications from subcutaneous therapy. They would also be considered in cases of foslevodopa intolerance.

## Data Availability

The raw data supporting the conclusions of this article will be made available by the authors, without undue reservation.
